# Supplementary figures and images for: Distribution of Porphyromonas gingivalis fimA and mfa1 fimbrial genotypes in subgingival plaques
Source: PeerJ. 2018 Aug 27;6:e5581. doi: 10.7717/peerj.5581 (PMC6118206; doi:10.7717/peerj.5581)

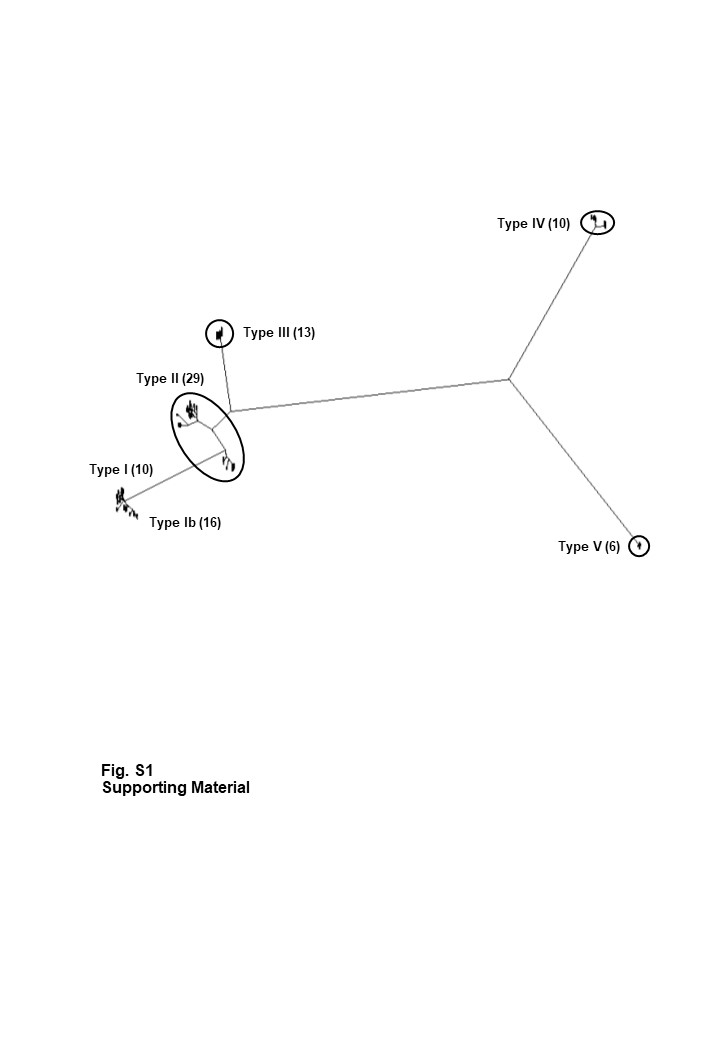

Supplement: Supplemental Information 1 — This is a modification of the phylogenetic tree from our previous study (Nagano et al., 2013); fimA genotypes and the number of strains analyzed (in parenthesis) are indicated at each node. The areas of genotypes I and Ib were not circled because the division between these genotypes is unclear. On the other hand, genotypes II and IV are obviously divided into two or more groups. [file peerj-06-5581-s001.jpg]
